# Effects of Porous Size and Membrane Pattern on Shear Stress Characteristic in Gut-on-a-Chip with Peristalsis Motion

**DOI:** 10.3390/mi14010022

**Published:** 2022-12-22

**Authors:** Pannasit Borwornpiyawat, Ekachai Juntasaro, Sasitorn Aueviriyavit, Varangrat Juntasaro, Witsaroot Sripumkhai, Pattaraluck Pattamang, Rattanawan Meananeatra, Kornphimol Kulthong, Ratjika Wongwanakul, Numfon Khemthongcharoen, Nithi Atthi, Wutthinan Jeamsaksiri

**Affiliations:** 1 Mechanical Engineering Simulation and Design Group, The Sirindhorn International Thai-German Graduate School of Engineering (TGGS), King Mongkut’s University of Technology North Bangkok (KMUTNB), Bangkok 10800, Thailand; 2 Nano Safety and Bioactivity Research Team, National Nanotechnology Center (NANOTEC), National Science and Technology Development Agency (NSTDA), Pathum Thani 12120, Thailand; 3 Department of Mechanical Engineering, Kasetsart University, Bangkok 10900, Thailand; 4 Thai Microelectronics Center (TMEC), National Electronics and Computer Technology Center (NECTEC), National Science and Technology Development Agency (NSTDA), Chacheongsao 24000, Thailand; 5 National Electronics and Computer Technology Center (NECTEC), National Science and Technology Development Agency (NSTDA), Pathum Thani 12120, Thailand

**Keywords:** porous membrane, shear stress, CFD, simulation, gut-on-a-chip, peristalsis motion

## Abstract

Dynamic gut-on-a-chip platform allows better recreation of the intestinal environment in vitro compared to the traditional static cell culture. However, the underlying mechanism is still not fully discovered. In this study, the shear stress behavior in a gut-on-a-chip device with porous membrane subjected to peristalsis motion is numerically investigated using CFD simulation for three different pore sizes and two pattern layouts. The results reveal that, in the stationary microchannel, the average shear stress on the porous membrane is approximately 15% greater than that of the flat membrane, regardless of the pore size. However, when subjected to cyclic deformation, the porous membrane with smaller pore size experiences stronger variation of shear stress which is ±5.61%, ±10.12% and ±34.45% from its average for the pore diameters of 10 μm, 5 μm and 1 μm, respectively. The shear stress distribution is more consistent in case of the staggered pattern layout while the in-line pattern layout allows for a 32% wider range of shear stress at the identical pore size during a cyclic deformation. These changes in the shear stress caused by peristalsis motion, porous size and membrane pattern could be the key factors that promote cell differentiation in the deforming gut-on-a-chip model.

## 1. Introduction

Intestines serve as major absorption sites for foods and drugs taken orally [1,2]. Caco-2 cells are generally used as in vitro model to estimate in vivo absorption, bioavailability, and first-pass metabolism as well as to study other effects on the intestinal epithelium [3,4,5]. Traditionally, cells are cultured on biocompatible surface of devices such as Petri dish or micro well plate with porous insert, i.e., Transwell [6]. However, cells cultured in these devices are in the static environment and usually differentiate into two-dimensional structures which inadequately represent the actual cell characteristic in vivo [7]. 

Over the past ten years, microfluidic technology has attracted scientists’ attention to incorporate it in biomedical applications [8,9,10]. The integration of cell culture into these micro-devices is collectively known as organ-on-a-chip, offering the ability to recreate the dynamic environment that allows key physiological functions of organs to be simulated in vitro [11,12,13]. Kim et al. (2012) reported that Caco-2 cells cultured in the gut-on-a-chip device rapidly developed and grew into multiple folds which recapitulated the structure of intestinal villi better than cells cultured in the static Transwell and could be cultured for a longer period of time. That was achieved by flowing the fluid into the microchannels, where cells were cultured within the chip, at 30 µL/h (8.333 × 10^6^ µm^3^/s) which generated the shear stress equal to 0.02 dyne/cm^2^ (0.002 Pa). The additional cyclic mechanical strain with the amplitude of 10% and the frequency of 0.15 Hz was applied to the porous membrane to mimic the peristalsis motion of the actual intestine [14]. Numerous studies also confirmed that intestinal cells cultured in the dynamic environment of gut-on-a-chip experienced better morphology, compared to the conventional cell culture [15,16,17,18]. Although the underlying mechanism still has not been fully understood until now [19], the shear stress of 0.02 dyne/cm^2^ became a reference value to maintain for Caco-2 cells cultured in many gut-on-a-chip applications recently. However, since the cells grow and differentiate during the polarization stage, it may cause the shear stress that is experienced by the cells to be different from that at the earlier stage [20]. Apart from this, the effect of cyclic deformation on the fluid flow inside the chip is believed to be negligible due to the small size of microchannels and the low frequency of periodically deforming membrane [21]. However, it is speculated that this might not be true because small disturbance to the fluid flow in a small passage may cause relatively large change in flow behavior, especially when it occurs repeatedly.

In Delon et al. (2019), the effect of shear stress on the morphology of Caco-2 cells was systematically investigated by utilizing a linear-shear-stress flow chamber. It was revealed that the cells reacted differently depending on the range of shear stress over 0.01–0.03 dyne/cm^2^. However, it should be noted that the study was conducted in a single-compartment PDMS microchannel bonded to a glass cover slip where the cells adhered to [22], while other gut-on-a-chip devices were generally composed of two-compartment microchannels separated by the porous membrane where the cells were cultured upon [14,15,16,17,18,23,24,25]. The fluid flow in the lower compartment of microchannel was reported to have an important role in the formation of the villi and three-dimensional structure of the intestinal epithelium [26]. Furthermore, the mechanical deformation of the epithelium was reported to help regulate the number of bacterium in the gut-on-a-chip [27]. Additionally, the shear stress distribution within the microchannel was reported to be affected by the peristalsis motion of the side-wall [28,29], as well as the microchannel dimensions and the membrane permeability [30]. However, these studies were conducted in two-dimensional space and relied heavily on mathematical formulations.

In this work, the effects of porous size and membrane pattern on the shear stress characteristic within a gut-on-a-chip device is systematically studied using three-dimensional CFD simulation with the dynamic mesh technique. The results are compared between the cases of (1) porous membrane and flat membrane, and (2) with and without the effect of side-wall peristalsis motion. Three pore sizes, i.e., 10, 5 and 1 µm, and two pattern layouts, i.e., staggered and in-line, are investigated. The results are discussed from the viewpoint of fluid mechanics as well as the cell culture perspective to establish a better understanding of the relationship between the shear stress and the cell characteristic. 

## 2. Materials and Methods

### 2.1. Governing Equations

Most organ-on-a-chip applications involve fluid flow at a very low flow rate within a small microchannel. This leads to a smooth flow regime known as laminar flow which is characterized by Reynolds number less than 2300 for internal flow [31]. Reynolds number can be calculated from,

(1) Re=ρVDhμ

where ρ is the fluid density, V is the mean flow velocity, $D_{h}$ is the hydraulic diameter of the flow passage and μ is the fluid dynamic viscosity. In this study, the problem is considered as 3D time-dependent incompressible flow and is governed by the Navier–Stokes equations as follows:

(2) $$\nabla \cdot \vec{u}=0$$

(3) $$\frac{\partial \vec{u}}{\partial t}+\left(\vec{u}\cdot \nabla \right)\vec{u}=-\frac{1}{\rho }\nabla p+\nu \nabla^{2}\vec{u}$$

where $\vec{u}$ is the velocity vector, t is the time, p is the pressure and ν is the kinematic viscosity of the fluid which can be related to the dynamic viscosity as,

(4) $$\nu =\frac{\mu }{\rho }$$

The shear stress can be determined from the product of the fluid dynamic viscosity and the shear strain rate at the point on the surface of interest as,

(5) $$\tau_{w}={\left.\mu \frac{\partial u}{\partial y}\right|}_{y=0}$$

For a specific case of flow in the rectangular microchannel, a simplified equation for calculating the shear stress is generally written in the following form [32,33,34]:

(6) $$\tau_{w}=\frac{6\mu Q}{{\mathrm{WH}}^{2}}$$

where Q is the volume flow rate, W is the width of microchannel and H is the height of microchannel. However, the shear stress obtained from Equation (6) is only the average shear stress in the microchannel and cannot account for the effect of a porous membrane on the shear stress. For such reasons, Equation (5) is used to calculate the shear stress and Equation (6) is used to determine the fluid dynamic viscosity in this study.

### 2.2. Numerical Modelling and Case Studies

**Figure 1:**
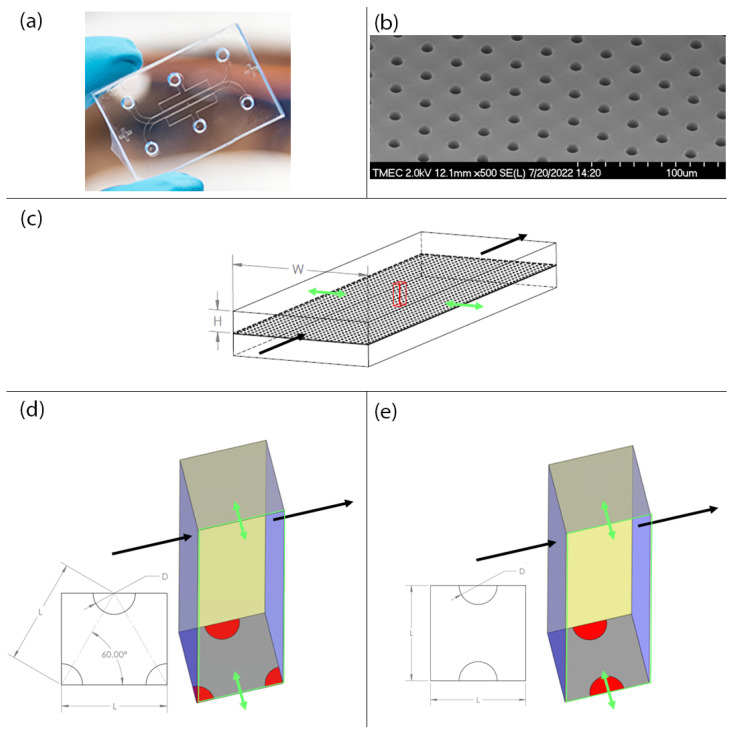
Pictures of the gut-on-a-chip prototype and illustrations of the computational domains. (**a**) The gut-on-a-chip prototype. (**b**) The electro microscopic image of the porous membrane in the gut-on-a-chip prototype. (**c**) The main microchannels in the gut-on-a-chip prototype with the block of computational domain shown in red. (**d**) The computational domain with the staggered porous membrane pattern. (**e**) The computational domain with the in-line porous membrane pattern. The black arrows indicate the direction of the main flow and the green arrows indicate the direction of peristalsis motion.

ANSYS Fluent (Academic version 2019R3) is used to perform the CFD simulation in order to investigate the effects of porous size and membrane pattern on shear stress characteristic within the gut-on-a-chip device. The computational domain, based on the gut-on-a-chip prototype in Figure 1a,b, is created by considering only one small portion of the upper microchannel as indicated by a red block with a square base, in which case the width, W, and the height, H, of the upper microchannel are equal to 1000 µm and 150 µm, respectively, as shown in Figure 1c. 

The computational domain surfaces are prescribed with the following boundary conditions. The left and right surfaces (blue) are prescribed with the periodic boundary condition. The top and bottom surfaces (grey) are set to the no-slip wall condition. The back side (yellow) is prescribed with the symmetry boundary condition. The front side (green outline) is prescribed with the symmetry boundary condition and also subjected to the peristalsis motion that stretches the domain in the direction perpendicular to the main flow forth and back (green arrows). The membrane is assumed to be very thin compared to the size of microchannel so that the membrane thickness is negligible and the symmetry boundary condition is applied to the circular sectors on the bottom surface (red) to mirror the presence of the flow in the lower microchannel. The total area of the circular sectors on the bottom surface of computational domain, i.e., the porous membrane, is equal to the area of a single circle as shown in Figure 1d,e.

**Figure 2:**
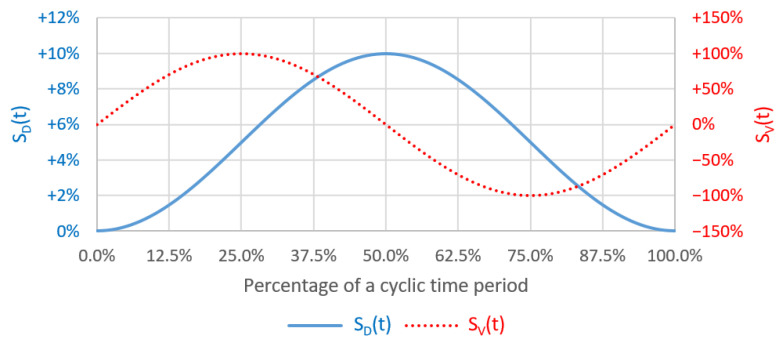
The plots of temporal displacement (blue) and velocity (red) of the front side in a deformation cycle.

In order to mimic the peristalsis motion, the front side is assumed to move as a rigid body and its motion is modeled by using the dynamic mesh user-defined function (UDF) as described by Equations (7) and (8) for its displacement and velocity, respectively. The temporal displacement and velocity of the front side in a deformation cycle is shown in Figure 2.

(7) $$S_{D}\left(t\right)=\frac{1}{2}\varepsilon \left(1-\cos\left(2\pi f\cdot t\right)\right)$$

(8) $$S_{V}\left(t\right)=\frac{\pi f\varepsilon \sin\left(2\pi f\cdot t\right)}{\max\left(\frac{{\mathrm{dS}}_{D}\left(t\right)}{\mathrm{dt}}\right)}$$

where ε is the maximum mechanical strain of the membrane and f is the frequency of the deformation cycle. In this study, ε=10% and f=0.15 Hz are adopted from Kim et al. (2012) which were reported to mimic the physiological peristalsis motion.

The actual dimension of the computational domain and the volume flow rate depend on the pattern of porous membrane in each case as summarized in Table 1. In this study, the fluid dynamic viscosity, μ, is calculated from Equation (6) using the parameters as reported in Kim et al. (2012), which is equal to 9.0 × 10^−4^ kg/m·s while the fluid density, ρ, is assumed to be equal to that of water at 37 °C, which is equal to 997.0 kg/m^3^ [35].

In order to calculate the average shear stress on the porous membrane, i.e., the bottom surface, as well as on the flat membrane, i.e., the top surface, the area-weighted average shear stress is computed by,

(9) $$\bar{\tau_{w}}=\frac{\sum_{i=1}^{n}\left(\tau_{w_{i}}\cdot A_{i}\right)}{\sum_{i=1}^{n}\left(A_{i}\right)}$$

where n is the number of control volumes at the surface of interest, $\tau_{w_{i}}$ is the shear stress of each individual control volume at the surface of interest, calculated from Equation (5), and $A_{i}$ is the surface area of each individual control volume at the surface of interest.

For Case no. 2, 4, 6 and 8 which are time-dependent, Equation (9) is used to compute $\bar{\tau_{w}}$ at every single time point in the simulation and, after the completion of each cycle, $\bar{\tau_{w}}$ is used to compute the time-averaged shear stress per cycle as follows:

(10) $$\overset{=}{\tau_{w}}=\frac{1}{T}\overset{N}{\underset{j=1}{\sum }}\left[\frac{\left({\bar{\tau_{w}}}_{j}+{\bar{\tau_{w}}}_{j-1}\right)}{2}\cdot \Delta t\right]$$

where N is the number of time steps in a cycle (N = 360), ${\bar{\tau_{w}}}_{j}$ and ${\bar{\tau_{w}}}_{j-1}$ are calculated from Equation (9) at the current and previous time points, respectively, Δt is the size of time step and T is the cycle time period. In this study, T=1/f=6.6667 s and Δt=T/N=0.0185 s.

### 2.3. Model Validation and Mesh Independent Analysis

**Figure 3:**
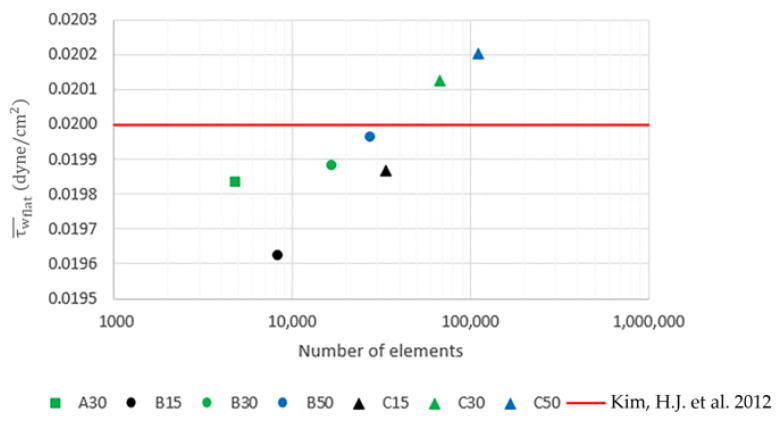
The plots of ${\bar{\tau_{w}}}_{\mathrm{flat}}$ obtained from different mesh levels [14].

The mesh independent analysis is carried out using Case no. 1. The number of elements used across the domain height is initially set to 30 elements and three different surface mesh levels, i.e., A, B and C, which are defined as the factors of the pore diameter, D, are used to perform the simulation to find the optimal size of the surface mesh. After that, the number of elements used across the domain height is decreased to 15 and increased to 50 in order to find the optimal mesh. The area-weighted average shear stress, $\bar{\tau_{w}}$, on the flat surface (top surface) is calculated and validated with the shear stress reported by Kim et al. (2012) [14] as summarized in Table 2. The area-weighted average shear stress on the flat surface obtained from different meshes are plotted against their corresponding number of elements as shown in Figure 3. Note that the simulations are performed until the residuals are less than 1.0 × 10^−6^ for all mesh levels.

According to the analysis, the mesh level B50 is selected because it has the smallest error. Since the size of surface mesh is defined as a factor of pore diameter, this way of mesh setup is thus applicable to all cases. However, in case of peristalsis motion, i.e., Case no. 2, 4, 6 and 8, the simulations are performed until the relative error of the time-averaged shear stress between the two consecutive cycles is less than 1%. This relative error is defined as,

(11) $$\%R.E.\left(\overset{=}{\tau_{w}}\right)=\left|\frac{{\overset{=}{\tau_{w}}}_{k}-{\overset{=}{\tau_{w}}}_{k-1}}{{\overset{=}{\tau_{w}}}_{k-1}}\right|\times 100\%$$

where k is the cycle number.

## 3. Results

### 3.1. Effect of Pore Size in the Stationary Microchannel

**Figure 4:**
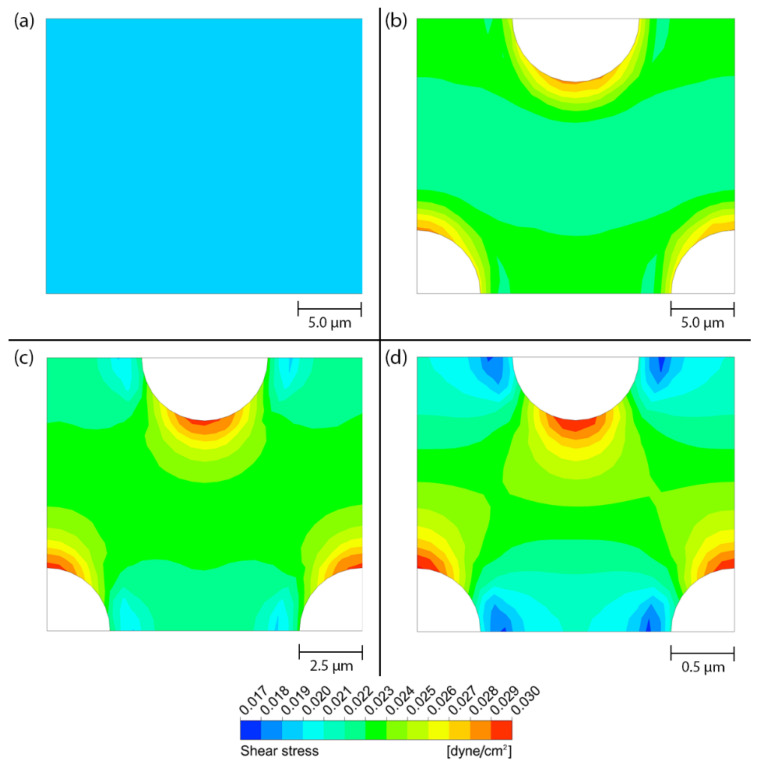
The contours of shear stress on different surfaces in the stationary microchannel. (**a**) Flat surface, (**b**) porous surface with the pore diameter of 10 µm, (**c**) porous surface with the pore diameter of 5 µm and (**d**) porous surface with the pore diameter of 1 µm.

Figure 4 shows the contours of shear stress on the flat surface and on the porous surface with different pore sizes (Case no. 1, 3 and 5), when the peristalsis motion is absent. In case of the flat surface, the area-weighted average shear stress, $\bar{\tau_{w}}$, is equal to 0.019965 dyne/cm^2^ and the result reveals that the shear stress is uniformly distributed across the entire surface as shown in Figure 4a. In case of the porous surface, the area-weighted average shear stress, $\bar{\tau_{w}}$, for the membrane with the pore diameters of 10 µm, 5 µm and 1 µm are equal to 0.023174, 0.023186 and 0.023190 dyne/cm^2^, respectively, which are approximately 15% greater than that of the flat surface. Although the area-weighted average shear stresses are almost identical for different pore sizes, the membrane with smaller pore diameter exhibits stronger shear stress variation across the surface compared to that of the larger pore diameter as shown in Figure 4b–d. 

**Figure 5:**
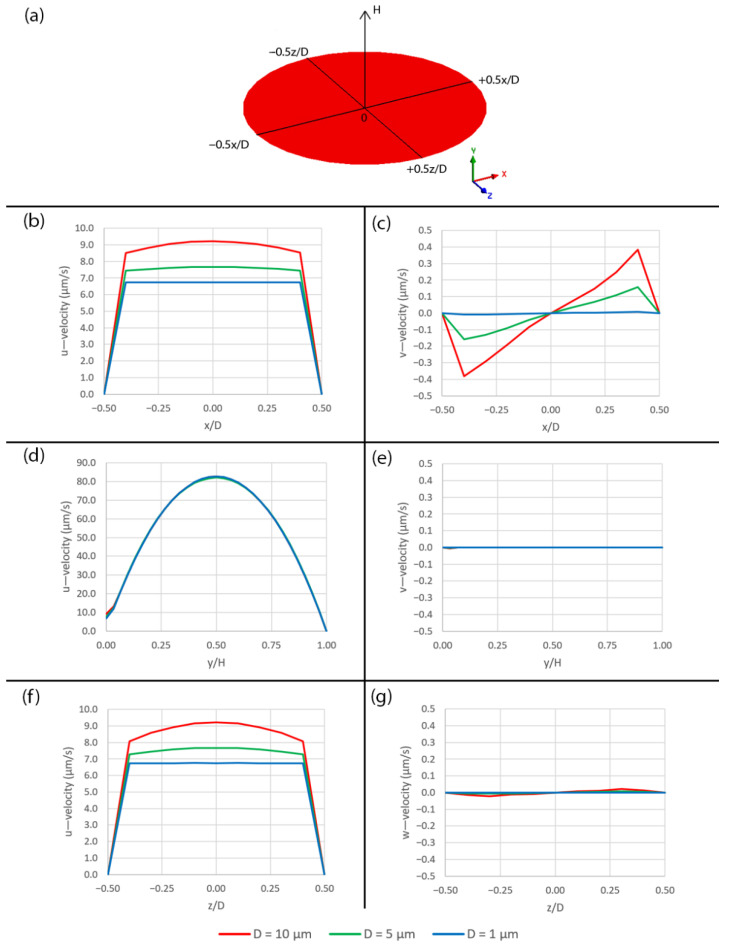
Diagram of plotting locations and plots of velocity components over the different pore sizes in case of no peristalsis motion. (**a**) The diagram, (**b**) plots of u—velocity along x-axis, (**c**) plots of v—velocity along x-axis, (**d**) plots of u—velocity along y-axis, (**e**) plots of v—velocity along y-axis, (**f**) plots of u—velocity along z-axis and (**g**) plots of w—velocity along z-axis.

The velocity components over the different pore sizes are plotted along the three axes as shown in Figure 5a. Along the x- and z-axes, the larger pore size allows higher velocity to develop across its opening, as shown in Figure 5b,c,f,g, because the smaller pore causes more friction along its wetted surface. Along the y-axis, the u—velocity has the parabolic profiles for all pore sizes, as shown in Figure 5d and the v—velocity is zero, as shown in Figure 5e. It should be noted that the u—velocity profiles in Figure 5d are not symmetrical because, while the flow sticks to the surface at y/H = 1, it slips over the pore at y/H = 0. Additionally, the w—velocity along the x- and y-axes as well as the v—velocity along the z-axis are zero due to the symmetry boundary condition.

**Figure 6:**
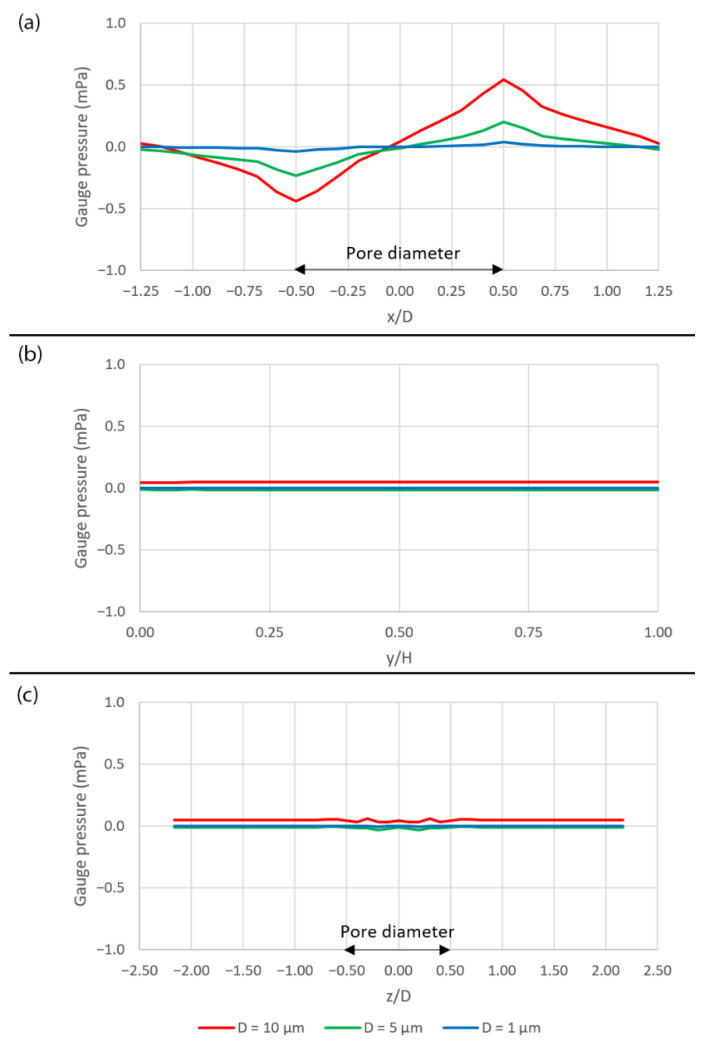
The plots of gauge pressure across the domain. (**a**) Along the x-axis, (**b**) along the y-axis and (**c**) along the z-axis. The plot locations are shown in Figure 5a.

Figure 6 shows the plots of pressure across the domain over the pore area. Along the x-axis, the pressure decreases as approaching the pore and then increases across the pore. After that, the pressure starts decreasing again, as shown in Figure 6a. The pore area with the negative pressure attracts the fluid into the pore while the pore area with the positive pressure drives the fluid out of the pore. This phenomenon can be seen clearly in Figure 5c where the v—velocity is negative over the first half of the pore and becomes positive over the second half which reflects the coupling between the pressure and the velocity, as described by the Navier–Stokes equations (Equation (3)). The pressure is constant along the y-axis as shown in Figure 6b and also along the z-axis, except over the pore area where there are some fluctuations, as shown in Figure 6c.

It should be noted that the pressure variation is stronger when the pore size is larger. Additionally, since the simulation is performed using the periodic boundary condition, instead of inlet and outlet, the pressure gradient across the domain is more important than the pressure itself. In case of no peristalsis motion, the pressure gradients across the domain are −26.401, −26.760 and −26.862 Pa/m for the case with pore diameters of 10 µm, 5 µm and 1 µm, respectively.

### 3.2. Shear Stress and Flow Behaviors in the Microchannel Subjected to Peristalsis Motion

**Figure 7:**
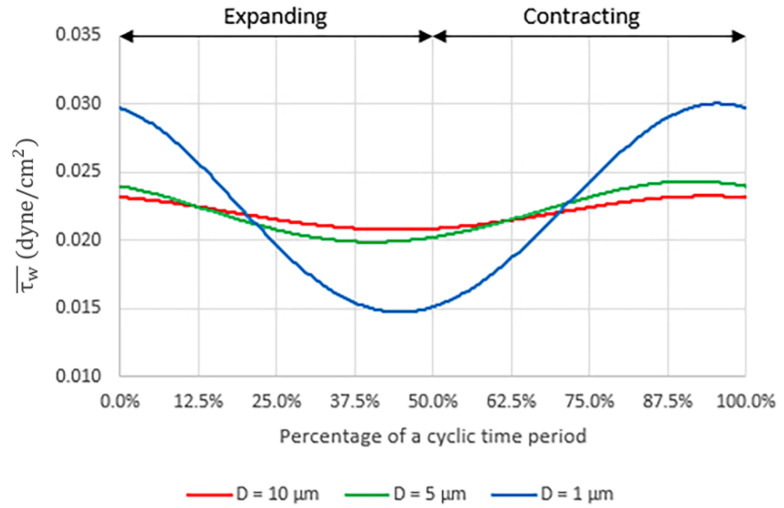
The plots of the area-weighted average shear stress, $\bar{\tau_{w}}$, on the porous surface with different pore sizes during a deformation cycle.

When peristalsis motion is present, the flow is disturbed by the cyclic deformation of the membrane which affects the shear stress behavior inside the microchannel. Figure 7 shows the plots of the area-weighted average shear stress, $\bar{\tau_{w}}$, on the porous surface with different pore sizes (Case no. 2, 4 and 6) during a deformation cycle. The time-averaged shear stresses per cycle, $\overset{\cdot }{\tau_{w}}$, are equal to 0.022003, 0.022052 and 0.022200 dyne/cm^2^ for the membrane with the pore diameters of 10 µm, 5 µm and 1 µm, respectively. Note that even though the time-averaged shear stresses are of the same magnitude in all three cases, the temporal variation is stronger with smaller pore diameter, that is, ±5.61%, ±10.12% and ±34.45% for the pore diameters of 10 µm, 5 µm and 1 µm, respectively.

**Figure 8:**
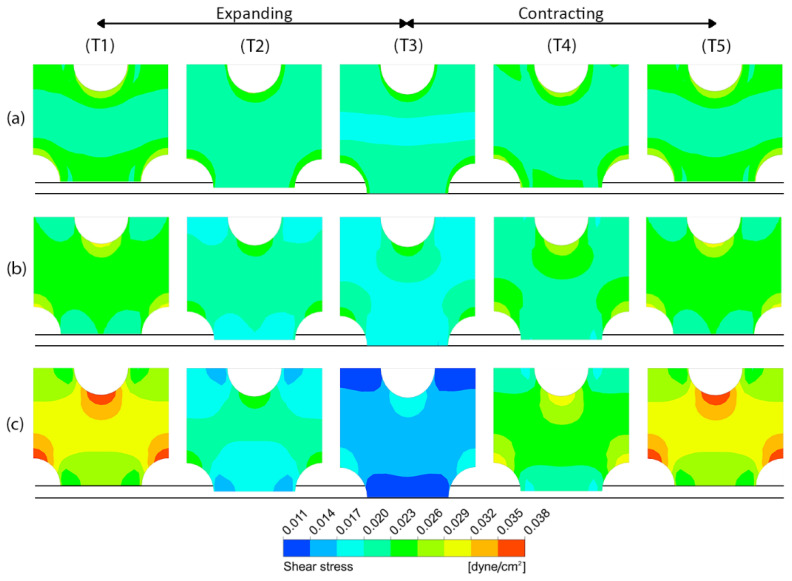
The contours of shear stress on the porous membrane with three different pore sizes at five different time points (T1–T5) during a deformation cycle. (**a**) D = 10 µm, (**b**) D = 5 µm and (**c**) D = 1 µm. The horizontal lines indicate the original position and the maximum deformation.

Figure 8 shows the contours of shear stress at five different time points (T1–T5) during a deformation cycle for different pore sizes. It is revealed that peristalsis motion helps expand the range of effective shear stress on the porous membrane surface, especially when the pore size becomes smaller. The shear stress ranges from 0.019678 to 0.028334 dyne/cm^2^ in case of the membrane with the pore diameter of 10 µm. For the membranes with the pore diameters of 5 µm and 1 µm, the shear stresses range from 0.017147 to 0.030191 dyne/cm^2^ and from 0.011246 to 0.038301 dyne/cm^2^, in which the ranges are approximately 50% and 200% larger compared to the membrane with the pore diameter of 10 µm, respectively.

**Figure 9:**
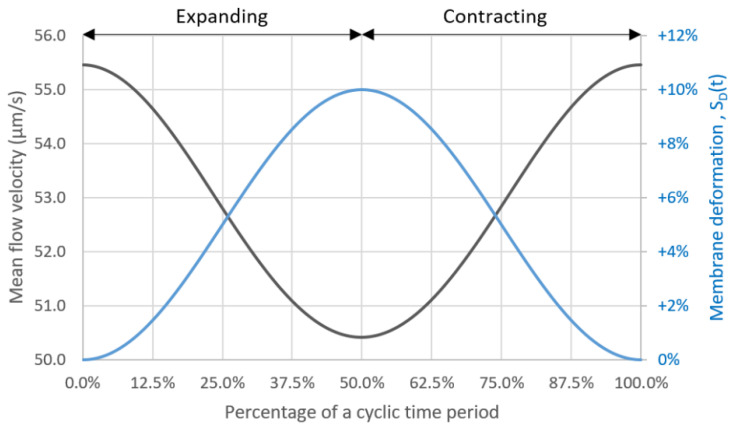
The plots of the mean flow velocity and the membrane deformation during a deformation cycle.

Furthermore, with the presence of peristalsis motion, the average shear stress is lower compared to that of the stationary microchannel. This is because the flow is temporarily drawn backward during the expansion phase which results in the lower mean flow velocity and hence the lower shear stress. The mean flow velocity is plotted together with the membrane deformation in a cycle as shown in Figure 9.

**Figure 10:**
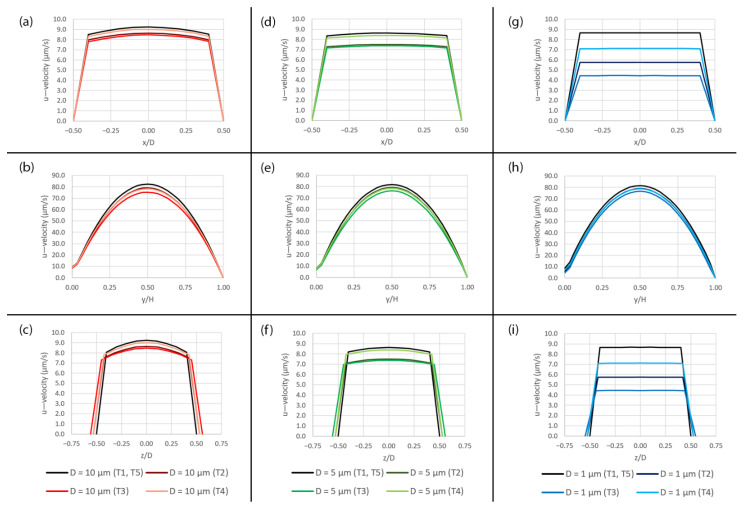
The plots of the u—velocity profile over the pore area subjected to peristalsis motion at five different time points (T1–T5) during a deformation cycle. (**a**) Along the x-axis over the 10 µm pore, (**b**) along the y-axis over the 10 µm pore, (**c**) along the z-axis over the 10 µm pore, (**d**) along the x-axis over the 5 µm pore, (**e**) along the y-axis over the 5 µm pore, (**f**) along the z-axis over the 5 µm pore, (**g**) along the x-axis over the 1 µm pore, (**h**) along the y-axis over the 1 µm pore and (**i**) along the z-axis over the 1 µm pore. The plot locations are shown in Figure 5a.

The primary component of the flow, i.e., the u—velocity, is plotted along the three axes over the pore area subjected to peristalsis motion at five different time points (T1–T5) during a deformation cycle as shown in Figure 10. It can be seen clearly that, along the x-and z-axes, the smaller pore has stronger velocity variation compared to the larger pore as shown in Figure 10a,c,d,f,g,i. The effect can also be seen at y/H = 0 although the overall velocity profiles are similar further away from the pore as shown in Figure 10b,e,h. The flow around the pore is affected by this behavior which results in the wider range of shear stress since it is calculated from the velocity gradient according to Equation (5). For the pore diameter of 1 µm, the profiles in Figure 10g,i, are almost flat because the pore is so small that the friction at its perimeter affects thoroughly across its opening.

**Figure 11:**
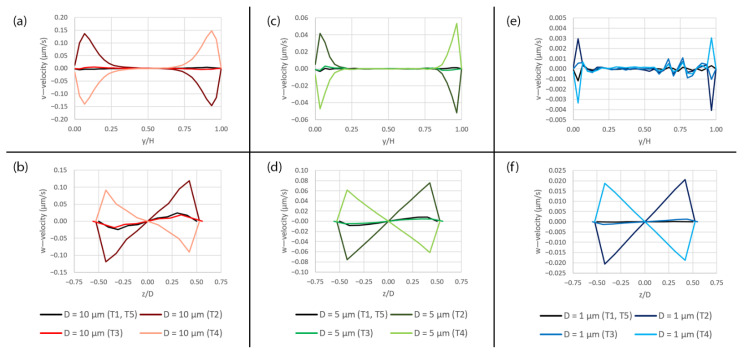
The plots of the v—velocity along the y-axis and the w—velocity along the z-axis over the pore area subjected to peristalsis motion at five different time points (T1–T5) during a deformation cycle. (**a**) Plots of the v—velocity along the y-axis over the 10 µm pore, (**b**) plots of the w—velocity along the z-axis over the 10 µm pore, (**c**) plots of the v—velocity along the y-axis over the 5 µm pore, (**d**) plots of the w—velocity along the z-axis over the 5 µm pore, (**e**) plots of the v—velocity along the y-axis over the 1 µm pore and (**f**) plots of the w—velocity along the z-axis over the 1 µm pore. The plot locations are shown in Figure 5a.

Additionally, the v—velocity along the y-axis and the w—velocity along the z-axis are plotted to show the secondary flow due to the deformation as shown in Figure 11. During the expansion (T2), the flow is partially drawn toward the mid plane between the upper and lower surfaces of the microchannel as indicated by the positive v—velocity at y/H < 0.50 and the negative v—velocity at y/H > 0.50 as shown in Figure 11a,c,e. At the same time, the flow is also drawn toward the side of the microchannel as indicated by the negative w—velocity at z/D < 0.00 and the positive w—velocity at z/D > 0.00 as shown in Figure 11b,d,f. On the contrary, the flow is in the opposite direction during the contraction (T4). Some fluctuations in the flow can be seen when the membrane is at the original position (T1 and T5) as well as at the maximum deformation (T3) which are caused by the fluid inertia.

**Figure 12:**
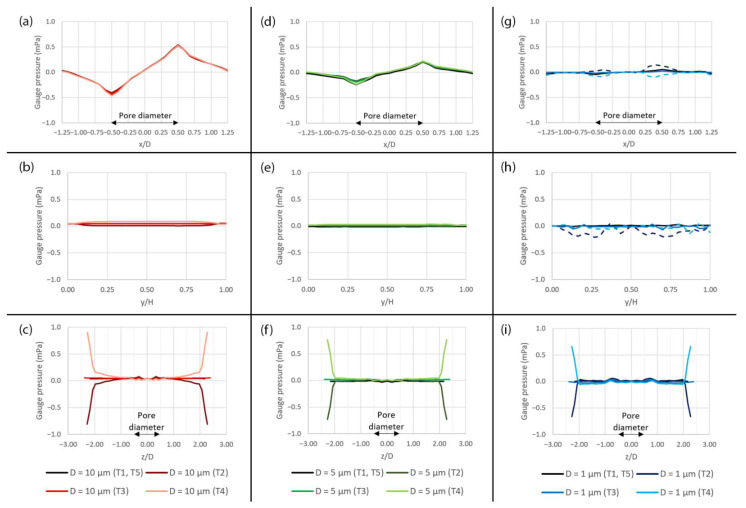
The plots of gauge pressure across the domain with different pore sizes at different time points (T1–T5) during a deformation cycle. (**a**) Along the x-axis over the 10 µm pore, (**b**) along the y-axis over the 10 µm pore, (**c**) along the z-axis over the 10 µm pore, (**d**) along the x-axis over the 5 µm pore, (**e**) along the y-axis over the 5 µm pore, (**f**) along the z-axis over the 5 µm pore, (**g**) along the x-axis over the 1 µm pore, (**h**) along the y-axis over the 1 µm pore and (**i**) along the z-axis over the 1 µm pore. The plot locations are shown in Figure 5a.

The profiles of pressure across the domain with different pore sizes at different time points (T1–T5) during a deformation cycle are shown in Figure 12. It is revealed that the changes in pressure during a deformation cycle is very small along both the x- and y-axes, as shown in Figure 12a,b,d,e,g,h. The profiles in these figures are similar to those of the flow without peristalsis motion, except some discrepancies that can be observed in case of the 1 µm pore during the expansion (T2) and the contraction (T4), as shown by the dashed lines. Along the z-axis, the pressure drastically decreases and increases near the side surfaces, i.e., |z/D| > 2.00, during the expansion (T2) and the contraction (T4), respectively, as shown in Figure 12c,f,i.

**Figure 13:**
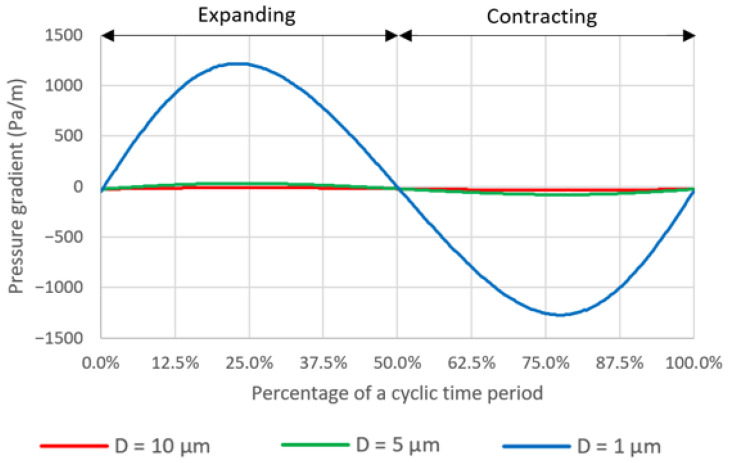
The temporal plots of pressure gradient across the domain during a deformation cycle.

The average pressure gradients during a deformation cycle are −25.019, −25.172 and −25.731 Pa/m for the case with pore diameters of 10 µm, 5 µm and 1 µm, respectively. The pressure gradient across the domain varies with time in a deformation cycle as shown in Figure 13. It should be noted that the magnitude of pressure gradient increases dramatically as the pore diameter decreases. It is speculated that this is because the simulation domains are scaled down, in both x- and z-axes, according to the pore diameters which are 2 times and 10 times smaller for the cases with the pore diameters of 5 µm and 1 µm, respectively, compared to the case with the pore diameter of 10 µm. The smaller domain can be described as a smaller flow passage which would have the higher flow resistance and thus require the greater driving force, i.e., pressure gradient, to push the fluid through. 

### 3.3. Effect of Pattern Layout on Shear Stress Distribution

**Figure 14:**
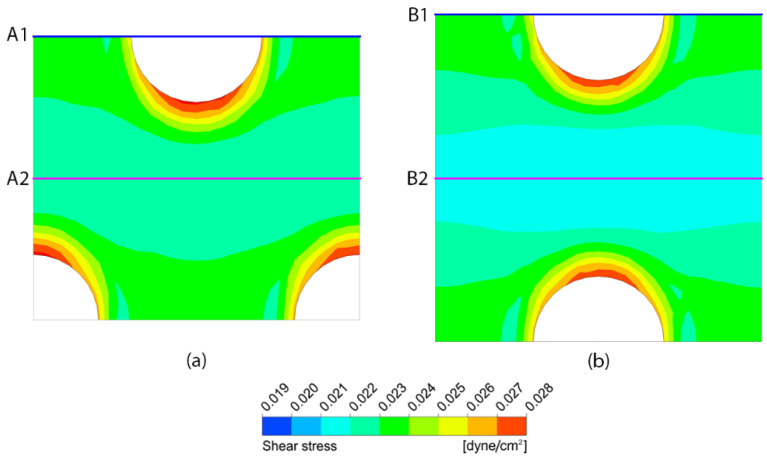
The contours of shear stress on the porous membrane with the pore diameter of 10 µm in case of (**a**) staggered pattern and (**b**) in-line pattern, in the stationary microchannel.

Figure 14 shows the difference between the shear stress distribution on the porous membrane surface with the pore diameter of 10 µm in case of the staggered pattern arrangement (Case no. 1) and that of the in-line pattern arrangement (Case no. 7), without peristalsis motion. The simulation results reveal that the area-weighted average shear stresses are 0.023174 and 0.022688 dyne/cm^2^ for the staggered pattern and the in-line pattern, respectively, where the difference is approximately 2%. The distributions of shear stress around the pore are similar in both cases. However, the contours of shear stress in the middle region between the pores are different. The contour in this region has a wavy pattern with higher shear stress in case of the staggered pattern, as shown in Figure 14a, while it has a bottleneck-like structure with lower shear stress in case of the in-line pattern, as shown in Figure 14b.

**Figure 15:**
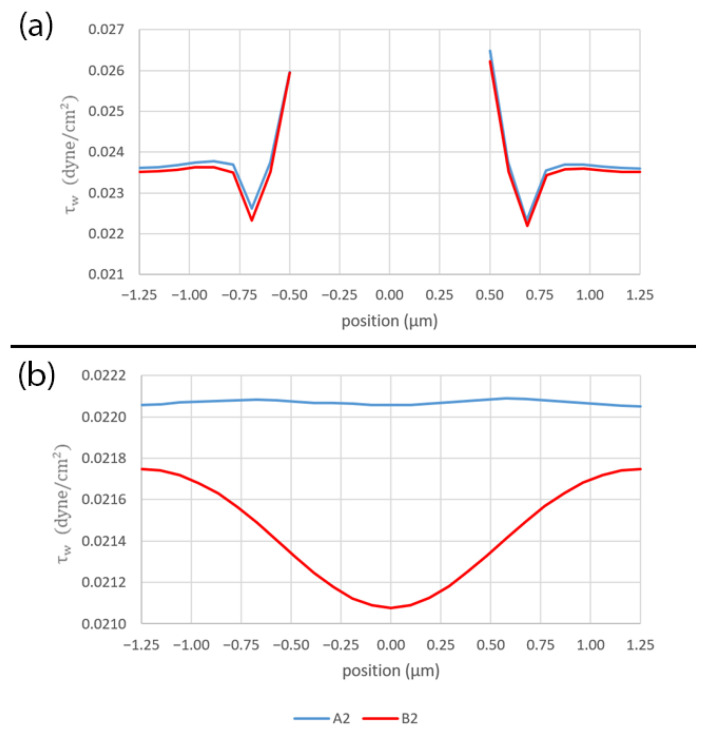
The plots of shear stress along the flow paths: (**a**) across the pore and (**b**) between the pores. The plot locations are shown in Figure 14.

Figure 15 shows the shear stress profiles along the flow paths: (a) across the pore (A1 and B1 lines in Figure 14) and (b) between the pores (A2 and B2 lines in Figure 14). Across the pore, the shear stress profiles are almost identical for both pattern layouts, as shown in Figure 15a. However, in the middle region between the pores, although the contour has a wavy pattern in case of the staggered pattern layout, the shear stress profile is almost constant along the A2 line at 0.022070 dyne/cm^2^. On the contrary, although the contour seems to suggest a linear profile, it has a stronger variation along the B2 line with the amplitude of ±0.000336 and the mean of 0.021433 dyne/cm^2^ in case of the in-line pattern layout, as shown in Figure 15b.

**Figure 16:**
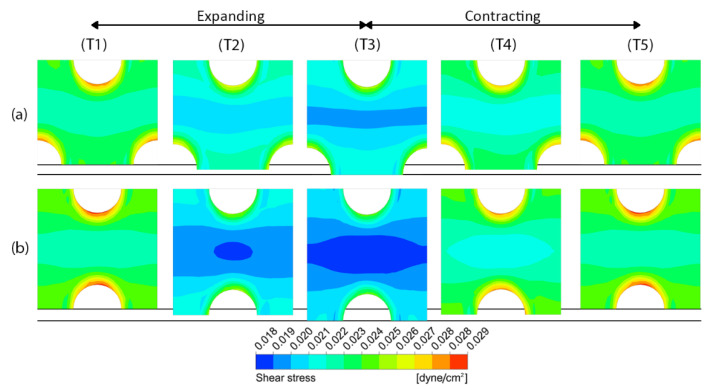
The contours of shear stress on the porous membrane with the pore diameter of 10 µm for two different pattern layouts at five different time points (T1–T5) during a deformation cycle. (**a**) The staggered pattern and (**b**) the in-line pattern. The horizontal lines indicate the original position and the maximum deformation.

During a deformation cycle, the porous membrane with the in-line pattern (Case no. 8) experiences a 32% wider range of shear stress across the surface compared to that of the staggered pattern (Case no. 2), in which the shear stress of the former ranges from 0.017954 to 0.029414 dyne/cm^2^ while the latter ranges from 0.019678 to 0.028334 dyne/cm^2^, as shown in Figure 16.

**Figure 17:**
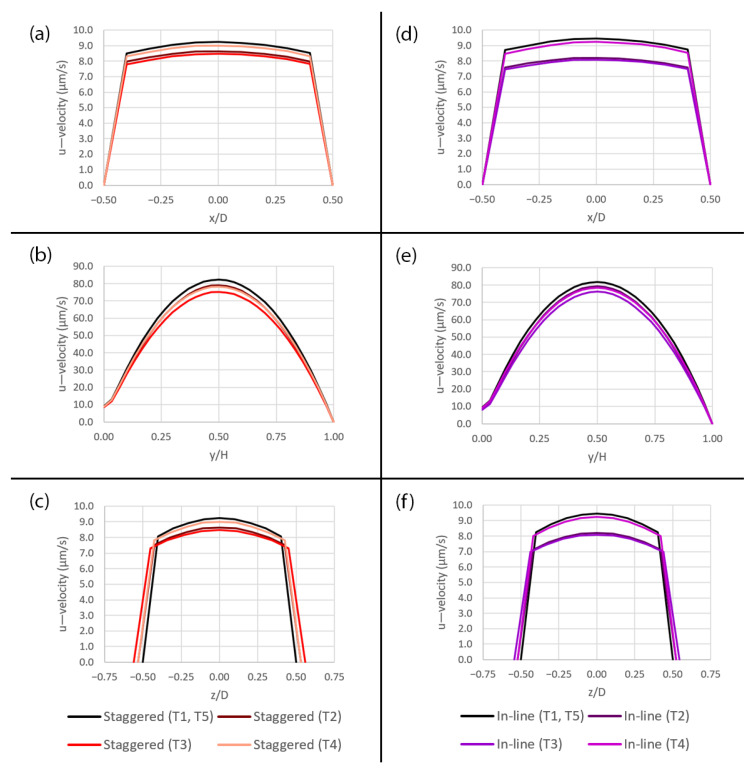
Plots of the u—velocity profiles over the pore with the diameter of 10 µm for two different pattern layouts at five different time points (T1–T5) during a deformation cycle. (**a**) Along the x-axis in the staggered pattern, (**b**) along the y-axis in the staggered pattern, (**c**) along the z-axis in the staggered pattern, (**d**) along the x-axis in the in-line pattern, (**e**) along the y-axis in the in-line pattern and (**f**) along the z-axis in the in-line pattern. The plot locations are shown in Figure 5a.

**Figure 18:**
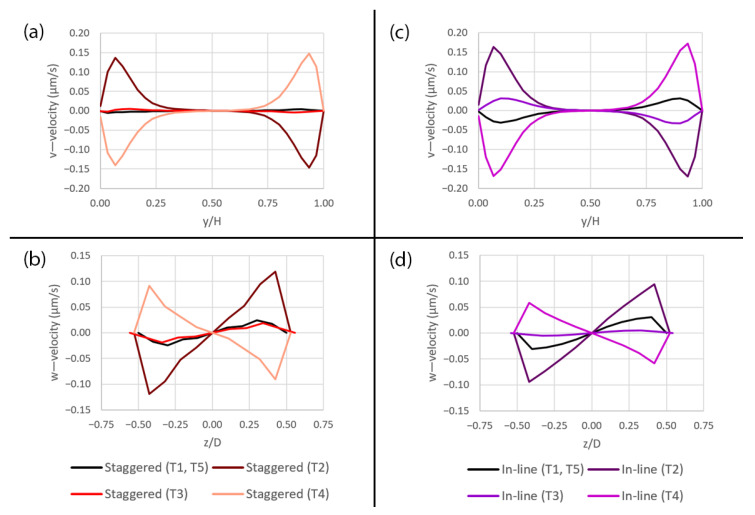
The plots of the v—velocity along the y-axis and the w—velocity along the z-axis over the pore with the diameter of 10 µm for two different pattern layouts at five different time points (T1–T5) during a deformation cycle. (**a**) Plots of the v—velocity along the y-axis in the staggered pattern, (**b**) plots of the w—velocity along the z-axis in the staggered pattern, (**c**) plots of the v—velocity along the y-axis in the in-line pattern and (**d**) plots of the w—velocity along the z-axis in the in-line pattern. The plot locations are shown in Figure 5a.

**Figure 19:**
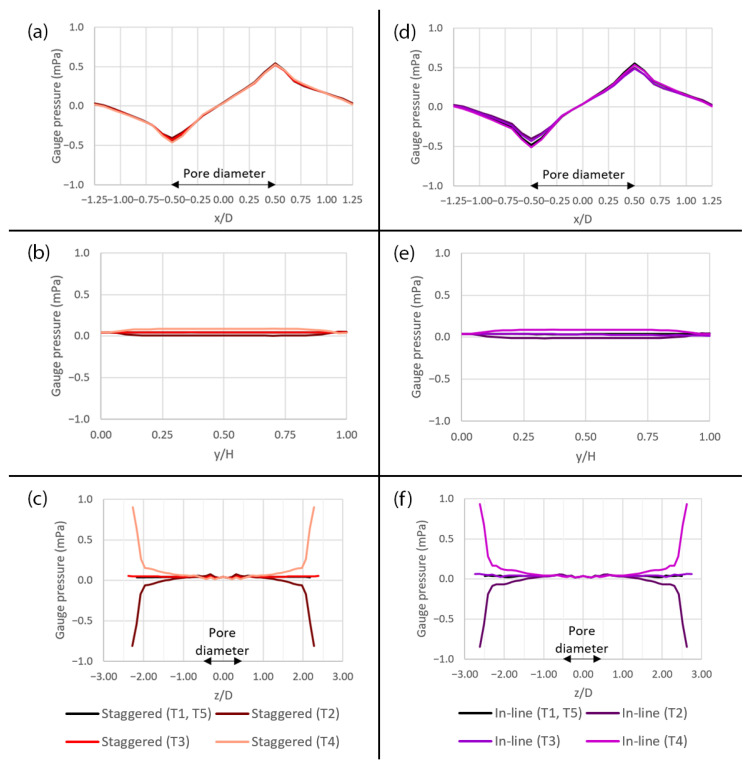
The plots of gauge pressure across the domain with the pore diameter of 10 µm for two different pattern layouts at five different time points (T1–T5) during a deformation cycle. (**a**) Along the x-axis in the staggered pattern, (**b**) along the y-axis in the staggered pattern, (**c**) along the z-axis in the staggered pattern, (**d**) along the x-axis in the in-line pattern, (**e**) along the y-axis in the in-line pattern and (**f**) along the z-axis in the in-line pattern. The plot locations are shown in Figure 5a.

**Figure 20:**
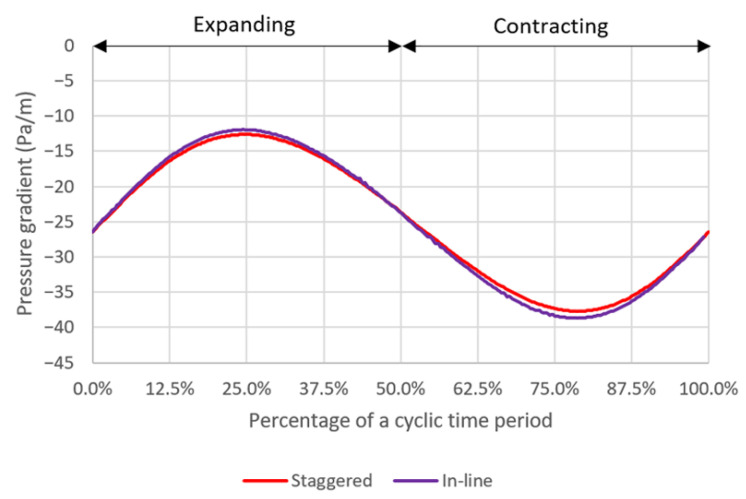
The temporal plots of pressure gradient across the domain for two different pattern layouts.

For both pattern layouts, the primary flow is visualized by the u—velocity along three axes as shown in Figure 17 and the secondary flow is visualized by the v—velocity along the y-axis and the w—velocity along the z-axis as shown in Figure 18. The pressure along three axes is shown in Figure 19 and the pressure gradient across the domain during a deformation cycle is shown in Figure 20. Overall, it is revealed that the profiles are very similar for both pattern layouts with a bit stronger variation in case of the in-line pattern. This is because the domain size in the z-direction, i.e., in the deformation direction, is greater which allows the flow to develop more than that of the staggered pattern, except that the w—velocity along the z-axis in Figure 18d has less variation in case of the in-line pattern because the distance between the pore centers along the z-axis is smaller and hence the flow cannot develop as much as that of the staggered pattern at this specific location.

## 4. Discussion

The simulation results reveal that the cells cultured on the porous membrane surface in the gut-on-a-chip device experiences a wide range of shear stress, from approximately 0.01 to 0.03 dyne/cm^2^ depending on the pore size and pattern layout, under the presence of peristalsis motion. According to Delon et al. (2019), Caco-2 cells cultured within the variable-width microchannel with the shear stress range from 0.01 to 0.03 dyne/cm^2^ have different characteristics, depending on the location and shear stress along the length of microchannel [22]. The present study shows that the similar range of shear stress can be achieved with a constant-width microchannel by exerting cyclic deformation on the membrane which affects the flow inside and causes the shear stress to periodically increase and decrease over time. This might be a key that makes the gut-on-a-chip device with cyclic deformation [14] so successful, since Caco-2 cells in the device would experience the highly dynamic range of shear stress throughout the culturing period.

The results also suggest that one might be able to directly control the flow inside the gut-on-a-chip device, without need to deform the membrane, in order to achieve the desired range of shear stress because the shear stress directly depends on the flow rate although the deformation of membrane might be another factor that contributes to recreating the appropriate environment within the gut-on-a-chip device [27]. The porous membrane with the fully confluent cell culture on its surface can be viewed as a thick flat membrane, although the apical surface of cell epithelium is not actually flat, that consumes a certain height in the microchannel and thus reduces the effective height of the flow passage. As a result, the apical surface would experience higher shear stress, according to Equation 6, depending on the epithelial height at the time. On the contrary, the basal surface would still expose to the same shear stress, depending on the pore size and pattern layout, as reported in this study. 

According to the present results, it is speculated that the staggered pattern with smaller pore size would be more preferable because it is revealed that the staggered pattern has more consistent shear stress distribution across the surface and, in the actual application, one would expect the cell culture to grow and differentiate evenly across the device. The smaller pore size would also help to enhance the attachment of the cells on the surface. Further investigation will be undertaken in order to see those effects on the actual cell culture.

It should be noted that even though the simulation is based on a gut-on-a-chip model, the same behavior can be expected for other organ-on-a-chip applications with the similar chip design with different ranges of shear stress obtained.

## 5. Conclusions

In the present study, the behavior of shear stress on the membrane surface in the gut-on-a-chip device is clearly and systematically investigated by using the CFD simulation with deforming mesh technique. The results reveal that the average shear stress on the porous membrane is approximately 15% greater than that of the flat membrane. When subjected to the peristalsis motion, the time-averaged shear stress is of the same magnitude for different pore sizes and layouts. However, the distribution and variation of local shear stress are different. The smaller pores experience stronger variation of shear stress than the larger pores during a deformation cycle. These variations of shear stress are ±5.61%, ±10.12% and ±34.45% for the porous membrane with the pore diameters of 10 μm, 5 μm and 1 μm, respectively. The in-line pattern has a 32% wider range of shear stress across the surface while the staggered pattern has more consistent distribution at the same pore size. The local distribution and cyclic variation of shear stress during the cell culturing period could be among the underlying mechanisms that help promote the cell differentiation in the gut-on-a-chip applications.

## Figures and Tables

**Table 1:** List of case studies with the corresponding parameters.

Case No.	Pattern Layout	Dimension ^1,2^ (µm)		Volume Flow Rate ^3^, Q (µm^3^/s)	Peristalsis Motion
		D	L		
1	Staggered	10.0	25.0	1.805 × 10^5^	No
2	Staggered	10.0	25.0	1.805 × 10^5^	Yes
3	Staggered	5.0	12.5	9.027 × 10^4^	No
4	Staggered	5.0	12.5	9.027 × 10^4^	Yes
5	Staggered	1.0	2.5	1.805 × 10^4^	No
6	Staggered	1.0	2.5	1.805 × 10^4^	Yes
7	In-line	10.0	25.0	2.083 × 10^5^	No
8	In-line	10.0	25.0	2.083 × 10^5^	Yes

^1^ The height, H, is fixed to 150 µm for all cases. ^2^ The porosity of porous membrane is constant in this study as the ratio of D/L is fixed. ^3^ Equivalent to 30 µL/h (8.333 × 10^6^ µm^3^/s) as reported in Kim et al. (2012).

**Table 2:** The different mesh levels with their corresponding number of elements and shear stress.

Mesh Level	Surface Meshing Factor	Number of Elements	Area-Weighted Average Shear Stress on Flat Surface, ${\bar{\tau_{w}}}_{flat}$ (dyne/cm^2^) ^1^	%Error from Reference
A30	10	4800	0.019834	−0.83%
B15	20	8280	0.019624	−1.88%
B30	20	16,560	0.019884	−0.58%
B50	20	27,600	0.019965	−0.18%
C15	40	33,420	0.019866	−0.67%
C30	40	66,840	0.020125	+0.63%
C50	40	111,400	0.020203	+1.01%
Reference	-	-	0.02 [14]	-

^1^ 1.0 dyne/cm^2^ = 0.1 Pa.

## Data Availability

Not applicable.
